# Effects of 10,000 steps a day on physical and mental health in overweight participants in a community setting: a preliminary study

**DOI:** 10.1590/bjpt-rbf.2014.0160

**Published:** 2016-06-16

**Authors:** Kornanong Yuenyongchaiwat

**Affiliations:** 1 Physiotherapy Department, Faculty of Allied Health Sciences, Thammasat University, Pathumthani, Thailand

**Keywords:** physical activity, physical health, profile of mood states, overweight, walking, physical therapy

## Abstract

**Background:**

Being overweight is associated not only with physical health problems, but also with risk of mental health problems. Increased physical activity (PA) has been recommended for the prevention of cardiovascular disease; however, little is known about the effect of walking on physical and mental health outcomes.

**Objective:**

The purpose of the study was to explore the effectiveness of a pedometer-based PA intervention on physical and mental health states.

**Method:**

Thirty-five overweight participants with body mass index (BMI) ≥25 kg•m^–2^ were selected and assigned to a 12-week pedometer-based walking program (10,000 steps•d^–1^). The profile of mood states, BMI, waist circumference (WC), body fat percentage (%BF), and lean body mass (LBM) were measured before and after the 12-week intervention. The number of step counts was recorded 5 days a week in a diary booklet.

**Results:**

The 30 participants who accumulated 10,000 steps•d^–1^ had significantly lower anxiety, depression, anger, fatigue, confusion, and total mood distress scores compared with measurements taken prior to the intervention. Further, the participants had higher vigor scores compared to baseline. Regarding physical health, the participants who accrued 10,000 steps a day had significantly lower body weight, WC, BMI, and %BP. After adjustment for gender, height, and daily steps at follow-up, changes in WC were negatively associated with depression, fatigue, confusion, and total mood distress.

**Conclusions:**

An increase in PA by accumulating at least 10,000 steps•d^–1^ over a 12-week period improves physical and mood states in sedentary, overweight individuals.

## BULLET POINTS

- To explore the effect of 10,000 steps a day in sedentary, overweight Thai subjects.
- 10,000 steps a day may help to decrease physical and mental health problems.
- They may also help to reduce risks of non-communicable disease and cardiovascular disease.

## Introduction

Recently, overweight and obesity present major health problems worldwide and these issues lead to non-communicable diseases (NCDs) and cardiovascular disease^1^ ^,^ ^2^. Further, evidence suggests that obesity is associated with mental health problems (e.g. depression), poor perceived health, low self-esteem, and body image concern^3^. There is also evidence that obese individuals can decrease mood disorders by controlling diet and body weight, managing stress, improving self-esteem, having psychological treatment, and exercising. Physical activity (PA) has been consistently linked to improved mood. In addition, there is strong evidence that PA helps to decrease anxiety and depression and improves mood, self-esteem, and body image^4^ ^-^ ^6^. Regarding the relationship between obesity and PA, several studies have shown that overweight/obesity was inversely related to PA. For example, high body mass index (BMI) and waist circumference (WC) are related to decreased PA^7^ ^,^ ^8^. A recent systematic review from 25 prospective cohort studies found that PA levels including low PA (e.g. walking less than 150 minutes/week) could prevent future depression^9^. Therefore, interventions to decrease mental and physical health problems by increasing PA should be considered.

Recently, programs that increase the number of daily walking steps have been promoted to improve PA and a pedometer or step-counting device has been commonly used to measure and promote PA. However, little research has been conducted on the effects of accumulating 10,000 steps with a pedometer on both physical and mood states. Therefore, the aim of this study was to determine the effect of 10,000 steps per day with a pedometer on the profile of mood states and physical health in overweight participants in a community setting.

## Method

### Participants and design

Thirty-five overweight adults aged 35-59 were recruited (BMI≥25 kg•m^–2^). The ethics and protocol were approved by the Ethics Committee of Thammasat University, Pathumthani, Thailand (approval number 069/2557). All participants gave written informed consent. The quasi-experimental study was designed to determine whether the effect of a pedometer-based intervention decreased mental and physical health problems.

### Measures and apparatus

Anthropometric measures, i.e. weight (kg), height (cm), and WC, were assessed prior to and after the intervention program. Evaluation of BMI was used as an indicator of being overweight. WC was measured at the level of the umbilicus. In addition, percentage of body fat mass (%BF), lean body mass (LBM; kg) were measured, which have been described in detail elsewhere^10^.

All participants completed the Profile of Mood States (POMS) scale^11^ to measure psychological well-being at baseline and 12 weeks after baseline assessment. The POMS is composed of 65 items rated on a 5-point Likert scale. The checklist items are comprised of 6 subscale scores: tension/anxiety, depression, anger/hostility, fatigue, confusion, and vigor. In addition, the total mood distress score was used to evaluate an overall measure of mood states. Subtraction of vigor scores from the sum of the negative mood (i.e. tension, depression, anger, fatigue, and confusion) was defined as the total mood distress. The POMS has been found to have good internal consistency of subscales, ranging from 0.63 to 0.96^11^.

The number of walking steps per day was measured at baseline with a pedometer (Yamax SW-200). The participants were asked to wear their sealed pedometer during the working day for 5 days whilst following their normal daily routine and then record the details in their booklet. During the 12-week intervention, the participants were instructed to walk at least 10,000 steps per day and record their steps in their diary. At the end of week 12, body weight, POMS, BMI, WC, WHR, %BF, and LBM were assessed again.

### Statistical analysis

Sample size calculations indicated that 30 participants were needed to complete the study to ensure sufficient power (80%) to detect an effect size (Cohen’s *d*=1.08). In order to allow for attrition, 35 overweight participants were recruited. Steps per day were calculated weekly (i.e. 5 working days per week). The change in the average number of steps per day over the 12-week intervention program was modeled for each participant. Descriptive data are presented as percentage (%), mean, and standard deviation (SD). Data were verified for normality of distribution (Kolmogorov-Smirnov goodness-of-fit test). Changes in physical health outcomes were calculated by subtracting physical health outcomes at follow-up from initial baseline physical health values. The paired t-test was used to determine whether the pedometer decreased significant changes in physical and mental health outcomes. Pearson’s correlations coefficients were calculated between POMS after a 12-week period and changed physical health. To examine the association between POMS and physical health outcomes after adjusting for possible confounding variables (e.g. gender, height, and daily walking steps at follow-up), partial correlational analysis was conducted. In addition, a regression analysis was performed to evaluate the utility of the effects of the pedometer for improving health outcomes in overweight participants. Multiple regression analyses were conducted to determine the unique contribution of 10,000 steps per day to the improvement of mental health outcomes after adjustment for initial POMS and daily walking steps. In the linear regression, the assumptions of normality of the residual scores were met. In addition, all analyses were conducted with SPSS version 20.0 and the level of statistical significance was considered as *p*<0.05.

## Results

### Changes in physical and mental health states with the pedometer-based intervention program

The mean age of the participants was 49.67±6.51 years. A total of 30 participants (9 males and 21 females) completed the walking program. Of the 5 participants (14.29%) who did not complete the protocol, one was pregnant and one had leg pain due to a herniated disc caused by heavy lifting. Based on the definition of PA by Tudor-Locke et al.^12^, the participants were defined as a sedentary (i.e. less than 5,000 steps a day) at baseline. As shown in Table 1, feelings of tension, depression, anger, confusion, fatigue, and total mood distress decreased significantly after the 12-week program, as did body weight, BMI, WC, and %BF. In addition, vigor scores increased significantly (*p*<.05 in all cases). In short, accumulating 10,000 steps a day resulted in a significant decrease in negative mood and negative physical health outcomes and significant improvements in vigor-activity with a large effect size (*d*≥.8 in all cases; Cohen^13^).

**Table 1:** Comparison between pre- and post-intervention programs in overweight participants.

	Pre-intervention Mean±SD	Post-intervention Mean±SD	Mean difference	95% confidence interval for the difference	*p*-value
Walking (steps/day)	4,540.53±1,959.00	10,500.20±2070.54	–5,960.67	–6774.85 to -5145.49	<.001
BW (kg)	71.40±10.73	69.87±10.33	1.53	.54 to 2.52	.004
WC (cm)	92.63±9.26	90.33±8.16	2.30	.80 to 3.80	.004
WHR	.88±.05	.89±.06	.01	–.01 to .03	.477
BMI (kg/m^2^)	27.86±4.33	27.25±3.93	.62	.18 to 1.06	.007
BF (%)	32.97±6.89	29.82±7.29	3.15	2.19 to 4.12	< 001
LBM (kg)	47.42±5.57	48.54±5.21	–1.12	–1.91 to -.34	.006

**Profile of Mood State Scores**					
Tension/Anxiety	16.13±4.46	13.37±3.73	2.77	.92 to 4.61	.005
Depression	22.53±7.78	19.97±5.67	2.57	.59 to 4.54	.013
Anger	19.40±5.88	17.60±5.35	1.80	.57 to 3.03	.005
Fatigue	11.73±4.86	11.06±4.86	.67	.14 to 1.20	.016
Confusion	13.37±4.87	12.33 ± 4.10	1.03	.16 to 1.91	.022
Vigor	23.80±4.37	27.33±3.63	–3.53	–4.64 to -2.43	<.001
Total mood distress	59.37±24.83	47.10±16.41	12.27	7.95 to 16.59	<.001

BW: body weight; WC: waist circumference; WHR: waist-hip ratio; BMI: body mass index; BF: body fat; LBM: lean body mass.

### Correlations between daily walking steps, profile mood states, and physical health outcomes at follow-up

As seen in Table 2, the participants who accrued 10,000 steps a day had a modest but significant inverse correlation with decreased body weight, BMI, and WC at 12 weeks. In addition, analysis of the correlation revealed that changes in WC were negatively associated with depression, fatigue, confusion, and total mood distress (*p*<.05 in all cases). Further, negative association was observed between %BF and anger state (*p*<.05; see Table 3). Overall, there was a large (r>0.4 in all cases, according to Cohen^13^) negative correlation between health outcomes (e.g. WC) and profile of mood states (e.g. depression).

**Table 2:** Bivariate correlation between daily walking steps and physical health at the 12-week follow-up.

Physical health outcomes	Daily walking steps at follow-up	
	Pearson correlation (*r*)	*p*-value
BW	–.46	.01
BMI	–.42	.02
WC	–45	.01
WHR	–.26	.16
%BF	–.30	.11
LBM	–.29	.12

BW: body weight; BMI: Body mass index; WC: waist circumference; WHR: waist-hip ratio; BF: body fat;

LBM: lean body mass.

**Table 3:** Bivariate correlations between physical health changes and profile mood states after a 12-week period.

POMS	Pearson correlation (*r*; *p*-value)					
	Δ BW	Δ WC	Δ WHR	Δ BMI	Δ %BF	Δ LBM
Tension	.01; .97	.06; .74	–.10; .61	.03; .86	.13; .51	–.12; .55
Depression	–.02; .91	–.37; .05	.01; .94	.00; 1.00	.18; .35	–.19; .32
Anger	.28; .14	–.32; .09	.16; .39	.27; .14	.43; .02	–.15; .44
Fatigue	.08; .66	–.41; .02	.17; .38	.09; .64	.24; .19	–.11; .56
Confusion	.08; .70	–.52; .00	.04; .85	.08; .67	.10; .62	–.02; .93
Vigor	.04; .85	–.24; .20	–.15; .42	.03; .88	.08; .67	–.04; .82
TMD	.12; .54	–.42; .02	.13; .51	.14; .48	.31; .10	–.17; .38

Δ: changes; POMS: profile of mood states; BW: body weight; WC: waist circumference; WHR: waist-hip ratio; BMI: body mass index; BF: body fat; LBM: lean body mass; TMD: total mood distress.

To determine whether the relationship between mental health states and physical health changes survived adjustment, partial correlation analysis was then computed adjusting for gender, height, and daily walking steps at follow-up.

Partial correlations showed that the relationships between changes in WC and depression, fatigue, confusion, and total mood distress survived adjustment for gender height and daily walking steps at follow-up. Further, the relationship between %BF and anger states also remained significant after adjusting for gender, height, and daily walking steps at follow-up (see Table 4). In other words, lower WC was still associated with lower depression, confusion, and total mood distress scores, after controlling for gender, height, and 10,000 steps a day. Moreover, the participants who had higher %BF were associated with higher anger states. In sum, the negative relationships between physical health and profile of mood states remained statistically significant, albeit modest, after controlling for gender, height, and 10,000 steps a day.

**Table 4:** Partial correlations between psychological well-being and physical health changes controlling for height, gender, and daily walking steps at follow-up among overweight participants.

POMS	Pearson correlation (*r*; *p*-value)					
	Δ BW	Δ WC	Δ WHR	Δ BMI	Δ %BF	Δ LBM
Tension	–.02; .94	.124; .54	–.049; .81	.01; .97	.05; .80	–.09; .64
Depression	.00; .99	–.449; .02	.035; .86	.03; .90	.18; .38	–.16; .41
Anger	.29; .15	–.302; .13	.200; .32	.28; .15	.45; .02	–.14; .47
Fatigue	.11; .58	–.407; .04	.193; .34	.12; .55	.29; .14	–.12; .56
Confusion	.11; 58	–.558; .00	.001; 1.00	.13; .53	.21; .29	–.06; .77
Vigor	.06; .77	–.203; .31	–.144; .47	.06; .78	.12; .55	–.06; .77
TMD	.13; .51	–.437; .02	.149; .46	.15; .45	.32; .10	–.16; .43

Δ: changes; POMS: profile of mood states; BW: body weight; WC: waist circumference; WHR: waist-hip ratio; BMI: body mass index; TMD: total mood distress; BF: body fat; LBM: lean body mass.

### Prediction of longitudinal changes in mental health problems over a 12 week later

In addition, the study examined whether the contribution of walking steps•d^–1^ to the prediction of follow-up mental health outcomes. As can be seen in Table 5, future depression scores were related to initial depression scores and daily walking steps at follow-up (*R* ^2^=.645, *p*<.001), and the more the number of walking steps (≥10,000 steps per day) or PA increased, the more the depression scores decreased.

**Table 5:** Multiple regression analysis effect of 10,000 steps per day on depression from baseline characteristics.

	Non-standardized coefficients (B)	Standardized Coefficients (β)	*p*-value	95% Confidence interval for B
Baseline depression scores	.54	.74	<.001	.36 to .72
Initial daily walking steps	–.00	–.32	.030	.00 to .00
Changed daily walking steps	–.00	–.31	.036	.00 to .00

## Discussion

The present study evaluated the efficacy of accumulating 10,000 steps per day on the mood states and physical health outcomes of overweight participants in a community setting. Further, these participants were leading a sedentary lifestyle (<5,000 steps per day)^12^. The main findings of the present study were that participants displayed decreased negative mood (i.e. anxiety, depression, anger, confusion, and total mood distress scores), body weight, BMI, WC, and %BF after increasing PA by accumulating ≥10,000 steps daily using the pedometer in a 12-week walking intervention. In addition, the results found that walking 10,000 steps a day played a role in decreasing depression scores after controlling for gender, height, initial depression scores, initial daily walking steps, and changes in daily walking steps.

The results confirm recent findings indicating that walking 10,000 steps per day is effective in increasing PA. In particular, a meta-analysis of 26 studies from 1966 to 2007 suggested that using a pedometer increased PA^14^. Further, the beneficial change in weight demonstrated in the current study agrees with other studies, which showed that 10,000 steps per day resulted in a significant decrease in body weight, BMI, %BF, and WC^15^ ^-^ ^17^. Further, Tudor-Locke^18^ described pedometer-based guidelines and cardiovascular health outcomes and concluded that increasing walking steps can improve BMI and cardiovascular health outcomes^18^. Thus, accumulating at least 10,000 steps per day may account, in part, for a reduction in WC and BMI.

In addition, the present study results indicate that walking 10,000 steps per day is effective in reducing negative mood states. Previous studies have also demonstrated positive effects following a walking intervention. Several systematic reviews have shown the effect of exercise (e.g. aerobic exercise, walking) on PA on emotional states (i.e. depression, anxiety, and mood)^4^ ^,^ ^9^ ^,^ ^19^ ^-^ ^21^. These reviews revealed that exercise/PA is related to decreased anxiety, depression, and mood states as well as improved psychological well-being.

In the present study, the benefits of accumulating 10,000 steps a day over a 12-week period were supported by the self-reported results of the POMS: decreased tension, depression, anger, confusion, fatigue, and total mood distress. Moreover, increased vitality was observed in the participants who attained 10,000 steps a day.

It has been known that the effects of exercise reduce stress; however, few studies have reported the accumulation of 10,000 steps a day specifically^22^ ^,^ ^23^. The American College of Sports Medicine (ACSM) and the American Heart Association (AHA) have recommended individual accumulations of at least 30 minutes of moderate-intensity PA (e.g. brisk walking) 5 days per week or 150 minutes per week for all population groups^24^. Other studies have shown that accumulating at least 10,000 steps•d^–1^ meets that minimum requirement^12^ ^,^ ^25^. Therefore, a daily target of 10,000 steps has been generally suggested to improve health outcomes in sedentary lifestyle and promote a decrease in negative mood (e.g. anxiety and depression).

With respect to POMS and physical outcomes, our results indicate that participants with decreased WC exhibited less depression, fatigue, confusion, and total mood distress scores after a 12-week intervention. In addition, the relationships between WC and negative mood disorders (i.e. depression, confusion, and total mood distress) were maintained after statistical adjustment for baseline walking steps, increased steps walk, gender, and age. Moreover, increasing steps daily (accumulating up to 10,000 steps a day) predicted future depression scores after controlling for initial depression scores and baseline daily walking steps. Therefore, the present study would suggest that one mechanism linking negative mood disorders with the 10,000 steps a day may involve changed physical outcome (i.e. decreased WC). In addition, WC is assumed to be a risk factor for obesity and an indicator for cardiovascular risk (e.g. type 2 diabetes, hypertension, cardiovascular disease)^2^. In a large survey with U.S. adults, Zhao et al.^26^ reported that WC is related to increased risk of depressive symptoms. Hence, the present study provides some supporting evidence for the involvement of 10,000 daily steps in participants with negative mood: they showed a decreased WC.

It should be noted that this study has a few limitations that may have affected the results. The absence of a control group is the most important limitation; therefore, the effects observed are overestimated, given that important confounders (i.e. placebo effects, polite patients, regression to the mean, recall bias, and natural history) were not controlled. Therefore, a high quality randomized controlled trial is strongly needed to confirm our results. The study had a relatively small sample size and most of the participants recruited were females (70%). Therefore, results cannot be used to draw conclusions for the whole population.

In summary, the use of a pedometer can improve PA, and the effect of walking with the goal of accumulating 10,000 steps per day results in improved POMS scores (i.e. decreased tension, depression, anger, fatigue, confusion, and total mood distress) as well as improved vigor in overweight adults with sedentary lifestyle.
